# Probiotics may not prevent the deterioration of necrotizing enterocolitis from stage I to II/III

**DOI:** 10.1186/s12887-019-1524-5

**Published:** 2019-06-08

**Authors:** Zheng-Li Wang, Li Liu, Xiao-Yu Hu, Lu Guo, Qiu-Yu Li, Yao An, Ya-Jun Jiang, Shi Chen, Xue-Qiu Wang, Yu He, Lu-Quan Li

**Affiliations:** 1 Department of Neonatal Diagnosis and Treatment Center, Children’s Hospital of Chongqing Medical University, No 136, Zhong shan 2 Road, Yuzhong district, Chongqing, 400014 People’s Republic of China; 2 Key Laboratory of Pediatrics in Chongqing, Chongqing, 400014 People’s Republic of China; 3 Chongqing International Science and Technology Cooperation Center for Child Development and Disorders, Chongqing, 400014 People’s Republic of China

**Keywords:** Intestinal diseases, Microbiota, Matched case-control study

## Abstract

**Background:**

Probiotic therapy can reduce the incidence of NEC. Therapeutic use of probiotics after NEC diagnosis reduces the severity of NEC in preterm infants or full-term infants is unclear. To evaluate the effect of probiotics on preventing the deterioration of necrotizing enterocolitis (NEC) from stage I to II/III.

**Methods:**

A retrospective matched cohort study was performed. Included patients were ultimately divided into two groups: the probiotic treatment group (probiotics were used ≥4 days) and the no probiotic treatment group. The differences in deterioration trends between the two groups were compared. Additionally, the risk factors associated with the deterioration of NEC were further analyzed with a case-control study.

**Results:**

A total of 231 infants met the inclusion criteria. Eighty-one pairs were matched according to similar gestational age and birth weight. Before matching, we found that the rate of deterioration of NEC from stage I to II/III in the group with probiotic treatment was similar to that in the group without probiotic treatment (23.1% [25/108] vs 26.0% [32/123], *P* = 0.614). After matching, the rate of deterioration of NEC between the two groups still had no significant difference (21.0% [17/81] vs 27.2% [22/81], *P* = 0.358). Logistic regression analysis showed that sepsis after NEC was an independent risk factor for NEC deteriorating from stage I to II/III (OR 2.378, 95% CI 1.005–5.628, *P* = 0.049).

**Conclusion:**

Probiotics may not prevent the deterioration of NEC from stage I to II/III in infants, but this conclusion should be treated with caution.

**Electronic supplementary material:**

The online version of this article (10.1186/s12887-019-1524-5) contains supplementary material, which is available to authorized users.

## Background

Necrotizing enterocolitis (NEC) is a serious gastrointestinal disease with a mortality rate reaching up to 20–30% [1, 2], and it has become an important cause of neonatal death. There have been no targeted treatment protocols for NEC, and we generally use symptomatic treatment. Infants with NEC commonly receive cessation of enteral nutrition, broad-spectrum antibiotics, gastrointestinal decompression and supportive treatment if necessary. Studies have suggested that prophylactic probiotic therapy can reduce the incidence of NEC in preterm infants [3–5]^,^ and meta-analyses have confirmed these findings [6, 7]. Studies of probiotic effects in term infants have focused on allergic diseases, immunomodulation, infectious and antibiotic-associated diarrhea, sepsis or growth [8]. Furthermore, approximately 10% of NEC cases occurred in full-term infants, but few studies have focused on this group of infants [9]. Compared with infants with stage I NEC, infants with stage II/III NEC, especially infants with stage III NEC, incur higher costs for hospitalization and care and have poorer prognoses [10]. However, until now, whether the therapeutic use of probiotics after NEC diagnosis reduces the severity of NEC in preterm infants or full-term infants is unclear. The aim of this study was to evaluate the effect of probiotic therapy on preventing the deterioration of NEC from stage I to stage II/III in infants.

## Methods

### Setting

The Department of Neonatology, Children’s Hospital of Chongqing Medical University, Chongqing, China, is a national clinical specialty department that currently has 300 beds and admits approximately 10,000 newborns each year.

### Data collection

A retrospective cohort study was conducted. Medical records were reviewed for all infants with stage I NEC who were admitted to the Children’s Hospital of Chongqing Medical University from January 2012 to March 2016. The clinical stage of NEC was defined according to the criteria that was originally proposed by Bell et al. [11] and the modified criteria subsequently reported by Walsh and Kriegman [12]. Stage I NEC was defined according to the presence of clinical signs such as temperature instability, gastric residuals, emesis, abdominal distension, occult blood in the stool (without fissure); further confirmation came through radiographic or sonographic findings of dilated intestinal tract with slight intestinal obstruction or normal intestine. Stage II NEC was defined according to the presence of clinical signs such as gross blood in the stool (without fissure), slight metabolic acidosis along with the clinical signs of stage I NEC and having radiographic or sonographic findings of pneumatosis intestinalis or portal vein gas. The criteria of stage III NEC included nonspecific clinical features such as hypotension, bradycardia and apnea in addition to the clinical features of stage II NEC and radiographic or sonographic findings of ascites or pneumoperitoneum.

**Fig. 1:**
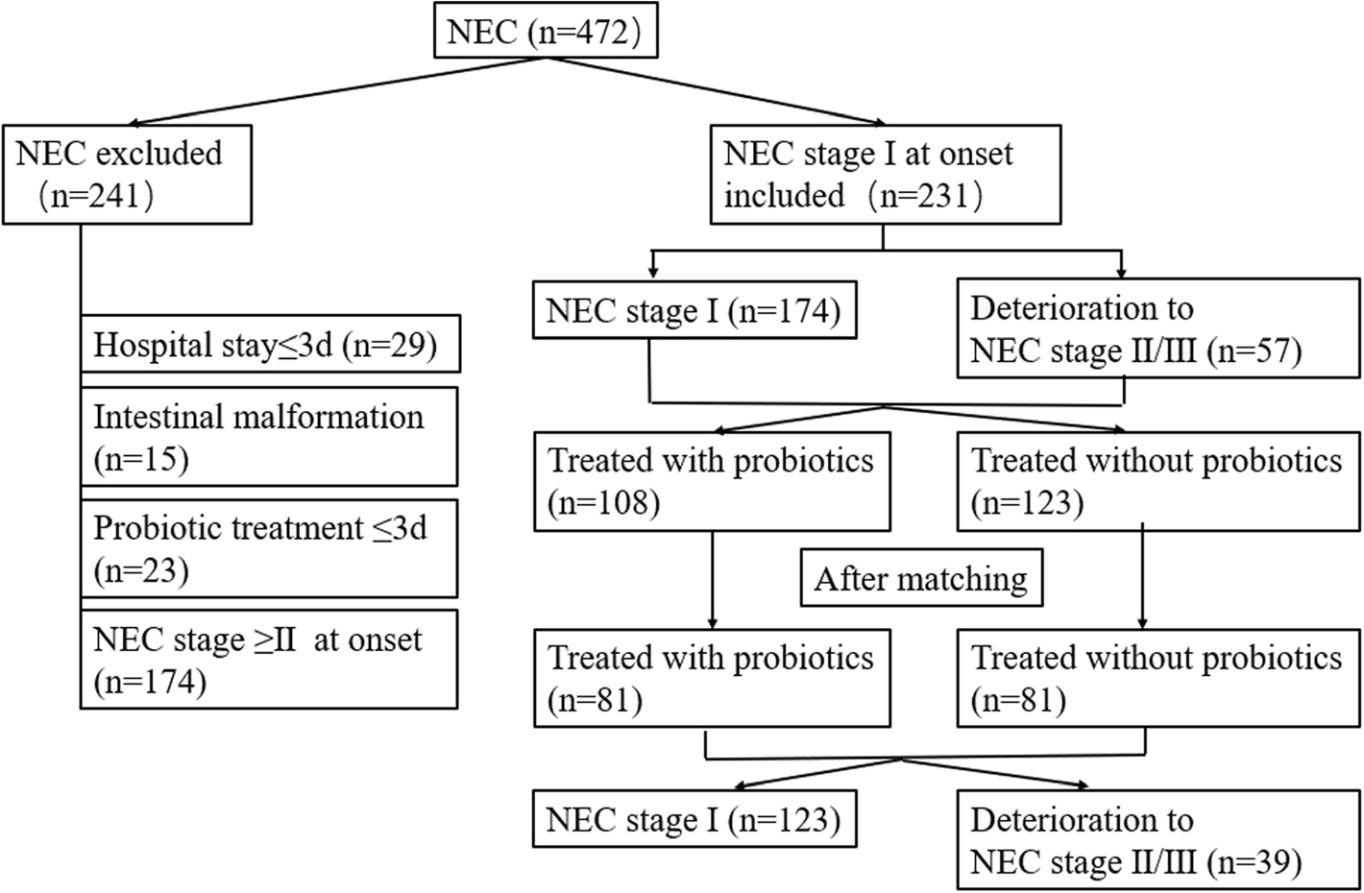
Flow chart for the study population and the subgroups

The age of NEC onset was defined as the day when one of the following signs or symptoms appeared: gastric residuals, emesis, abdominal distension, bloody stool (without fissure), diarrhea, or hypoactive bowel sounds. Patients with stage ≥II NEC at admission or with intestinal malformation (intestinal malrotation, intestinal stricture, intestinal atresia, Hirschsprung disease, anal atresia) or patients whose duration of hospitalization was ≤3 days were excluded from the study. Patients who received probiotics for ≤3 days were also excluded from further study (Fig. 1).

### Probiotic and other treatments

When stage I NEC was diagnosed, all patients included in this study received similar treatment protocols including cessation of enteral nutrition, total parental nutrition support, broad-spectrum antibiotic therapy and gastrointestinal decompression. For infants whose conditions deteriorated to stage II/III NEC, intensive care, including cardiorespiratory support and blood or blood product transfusion, was also provided when necessary.

Continuous clinical evaluation for infants and abdominal X-ray, full blood test (including white blood cells, platelet counts, immature/total neutrophils, etc.), C-reactive protein and procalcitonin examination were performed. If infants’ clinical conditions improved 72 h after diagnosis of NEC Bell stage I, enteral feeding was initiated (starting with 10 ml/kg of body weight formula milk, and then adding 10–20 ml/kg of body weight per day). When the patients were permitted to be fed, the infants in the probiotic group were treated with probiotics (≥4 days, one tablet at a time, bid), and the other infants in the control group were not treated with probiotics. The probiotics used in this study were *Bifidobacterium* tetravaccine tablets (live) [trade name: Hangzhou Longda Xinke Biological Pharmaceutical Co., Ltd., 0.5 g/tablet] with no less than 0.5 × 10^6^ colony-forming units (cfu) *infant Bifidobacteria*, *Lactobacillus acidophilus* and *Enterococcus faecalis,* 0.5 × 10^5^ cfu *Bacillus cereus.* The antenatal information, basic information, complications and treatment protocols of all patients included in this study were reviewed and compared between the two groups.

### Pairing method

To avoid attributing probiotics as the cause of advanced NEC stage when it may actually be attributable to gestational age and birth weight, a matched design was employed in our study. We performed 1:1 matching according to close gestational age (difference was ≤3 days) and birth weight (difference was ≤400 g). We first conducted a matched cohort study to identify whether the probiotic had an effect on the prevention of the deterioration of NEC from stage I to II/III. Then, we further explored the risk factors that might play an important role in deterioration of NEC from stage I to II/III by a case-control study. The details of the grouping are shown in Fig. 1.

### Statistical analysis

All data were analyzed by using SPSS 19.0 (SPSS Inc. Chicago, IL), and continuous variables were tested for normality using the Kolmogorov-Smirnov test. Normally distributed continuous data were described as the mean ± standard deviation (M ± S.D.) and were analyzed by *t* test. Skewed data were described as median (interquartile range, IQR) and were analyzed by Mann-Whitney *U* test. Categorical data were analyzed by using the Chi-square test or Fisher exact test. These statistically significant variables were tested again by logistic regression analysis to identify the independent risk factor. Statistical significance was established if *P* < 0.05.

## Results

### Patient demographics

In the study, 472 infants with NEC were admitted to the Children’s Hospital of Chongqing Medical University. A total of 231 infants met the inclusion criteria for further study, and 241 infants were excluded due to not meeting the criteria (hospitalization less than 3 days in 29 infants, 23 infants using 1–3 days of probiotics, 15 infants with intestinal malformation, 174 infants with stage ≥II at admission, and 8 infants with incomplete information).

The baseline characteristics of the infants are shown in Table 1. The median gestational age and birth weight of the patients were 37.86 weeks and 2800 g, respectively. Moreover, 39.4%(91/231) of the patients were premature infants. All infants with NEC were fed by formula during hospitalization. Overall, 24.7% (57/231) of infants progressed to stage ≥II during hospitalization. Before matching, differences in gestational age (*P* = 0.025), birth weight (*P* = 0.015), premature birth (*P* = 0.044), cortical steroid use in pregnancy (*P* = 0.047), anemia (*P* = 0.003) and gastrointestinal decompression (*P* = 0.048) were found between the probiotics group (*n* = 108) and the nonprobiotics group (*n* = 123). After matching, there were 81 pairs of infants who met the matching requirements, and no significant differences in these variables were found between the matched groups (*P* > 0.05).

**Table 1:** The baseline information of NEC infants treated (with/without) probiotics in this study

Variables	Total (*n* = 231)	Before matching			After matching^a^		
		With (*n* = 108)	Without (*n* = 123)	*P*	With (*n* = 81)	Without (*n* = 81)	*P*
Gestational age, IQR, wks	37.86 (34.57–39.71)	37.07 (34.04–39.25)	38.43 (35.14–39.86)	0.025	37.29 (34.71–38.86)	38.14 (34.86–39.50)	0.128
Premature, %(n)	39.4 (91)	46.3 (50)	33.3 (41)	0.044	42.0 (34)	35.8 (29)	0.420
Birth weight, (±SD), g	2800 (2000–3305)	2620 (1927.5–3180)	3000 (2100–3400)	0.015	2591.81 ± 718.45	2705.19 ± 713.69	0.315
Male, %(n)	40.7 (94)	48.9 (46)	51.1 (48)	0.582	47.1 (32)	52.9 (36)	0.524
Vaginal delivery, %(n)	42.0 (97)	38.0 (41)	45.5 (56)	0.245	39.5 (32)	46.9 (38)	0.341
The age of onset, IQR, d	3.44 (1.1–11.1)	5.49 (1.33–11)	3.14 (1–13)	0.355	5.57 (1.1–10.1)	3.4 (1–13.5)	0.988
PROM> 18 h, %(n)	5.2 (12)	2.8 (3)	7.3 (9)	0.121	2.5 (2)	6.2 (5)	0.440
Amniotic fluid contamination, %(n)	13.4 (31)	13.9 (15)	13.0 (16)	0.845	16.0 (13)	12.3 (10)	0.499
Asphyxia, %(n)	10.8 (25)	9.3 (10)	12.2 (15)	0.474	11.1 (9)	13.6 (11)	0.633
Infants of diabetic mothers, %(n)	2.6 (6)	3.7 (4)	1.6 (2)	0.565	3.7 (3)	2.5 (2)	1.000
PIH, %(n)	5.6 (13)	8.3 (9)	3.3 (4)	0.095	8.6 (7)	4.9 (4)	0.349
ICP, %(n)	2.2 (5)	2.8 (3)	1.6 (2)	0.883	2.5 (2)	1.2 (1)	1.000
Antenatal corticosteroids, %(n)	3.5 (8)	6.5 (7)	0.8 (1)	0.047	6.2 (5)	1.2 (1)	0.212

*IQR* interquartile range, *PROM* prolonged rupture of membranes, *PIH* pregnancy-induced hypertension, *ICP* intrahepatic cholestasis of pregnancy

^a^We performed 1:1 matching according to close gestational age (difference was ≤3 days) and birth weight (difference was ≤400 g)

The complications are shown in Table 2 and Additional file 1: Table S1. Several complications such as anemia (68.8%), coagulation disorder (59.3%), hypoproteinemia (51.9%), sepsis (31.2%), hypokalemia (32.5%), thrombocytopenia (21.2%) and respiratory failure (16%) were found in NEC infants. With the exception of anemia, the main complications had no significant difference between the two groups before or after matching (*P*>0.05).

**Table 2:** The complications or comorbidities of NEC infants treated (with/without) probiotics in this study

Variables	Total (*n* = 231)	Before matching			After matching^a^		
		With (*n* = 108)	Without (*n* = 123)	*P*	With (*n* = 81)	Without (*n* = 81)	*P*
NRDS, %(n)	7.4 (17)	8.3 (9)	6.5 (8)	0.595	7.4 (6)	6.2 (5)	0.755
Apnea, %(n)	6.1 (14)	8.3 (9)	4.1 (5)	0.175	6.2 (5)	3.7 (3)	0.717
Respiratory failure, %(n)	16.0 (37)	14.8 (16)	17.1 (21)	0.641	13.6 (11)	16.0 (13)	0.658
Pulmonary hemorrhage, %(n)	5.2 (12)	4.6 (5)	5.7 (7)	0.717	4.9 (4)	7.4 (6)	0.514
Sepsis, %(n)	31.2 (72)	37.0 (40)	26.0 (32)	0.071	39.5 (32)	32.1 (26)	0.325
Septic shock, %(n)	2.6 (6)	1.9 (2)	3.3 (4)	0.552	2.5 (2)	4.9 (4)	0.677
Bacterial meningitis, %(n)	4.3 (10)	3.7 (4)	4.9 (6)	0.910	3.7 (3)	7.4 (6)	0.493
Congenital heart disease^b^, %(n)	44.2 (102)	45.4 (49)	43.1 (53)	0.728	46.9 (38)	46.9 (38)	1.000
Cardiac insufficiency, %(n)	1.3 (3)	1.9 (2)	0.8 (1)	0.910	1.2 (1)	1.2 (1)	1.000
MODS,%(n)	0.4 (1)	0 (0)	0.8 (1)	1.000^a^	0 (0)	1.2 (1)	1.000^a^
Anemia, %(n)	68.8 (159)	79.6 (86)	59.3 (73)	0.001	76.5 (62)	60.5 (49)	0.028
Coagulation disorder, %(n)	59.3 (137)	63.9 (69)	55.3 (68)	0.184	61.7 (50)	55.6 (45)	0.425
Thrombocytopenia, %(n)	21.2 (49)	22.2 (24)	20.3 (25)	0.725	23.5 (19)	23.5 (19)	1.000

*NRDS* neonatal respiratory distress syndrome, *MODS* multiple organ dysfunction syndrome

^a^We performed 1:1 matching according to close gestational age (difference was ≤3 days) and birth weight (difference was ≤400 g)

^b^The congenital cardiac lesion: patent ductus arteriosus, ventricular septal defect or atrial septal defect. No special intervention was required in all cases after consultation with cardiologists and cardiac surgeons

The treatment protocols for NEC are shown in Table 3. Gastrointestinal decompression (64.1%), blood transfusion (23.4%), and dopamine support (25.5%) were also used for these NEC infants. Before matching, most treatment protocols had no significant difference between the two groups, with the exception of gastrointestinal decompression (*P* = 0.048). After matching, no difference in therapy was found between the two groups.

**Table 3:** The treatment protocols of NEC infants treated (with/without) probiotics in this study

Variables	Total (*n* = 231)	Before matching			After matching^a^		
		With (*n* = 108)	Without (*n* = 123)	*P*	With (*n* = 81)	Without (*n* = 81)	*P*
Gastrointestinal decompression, %(n)	64.1 (148)	57.4 (62)	69.9 (86)	0.048	58.0 (47)	67.9 (55)	0.193
Duration of gastrointestinal decompression, IQR, d	4 (2–6)	3 (2–6)	4 (2–6)	0.319	3 (2–6)	6 (2–6)	0.643
Red blood cell transfusion, %(n)	23.4 (54)	26.9 (29)	20.3 (25)	0.242	22.2 (18)	23.5 (19)	0.852
Platelet support, %(n)	6.5 (15)	7.4 (8)	5.7 (7)	0.597	8.6 (7)	7.4 (6)	0.772
Plasma support, %(n)	13.0 (30)	12.0 (13)	13.8 (17)	0.687	12.3 (10)	13.6 (11)	0.815
Intravenous immunoglobulin, %(n)	13.4 (31)	16.7 (18)	10.6 (13)	0.175	14.8 (12)	12.3 (10)	0.646
Albumin support, %(n)	43.7 (101)	41.7 (45)	45.5 (56)	0.555	38.3 (31)	44.4 (36)	0.425
Dopamine support, %(n)	25.5 (59)	25.0 (27)	26.0 (32)	0.860	23.5 (19)	24.7 (20)	0.854
Caffeine support, %(n)	9.1 (21)	9.3 (10)	8.9 (11)	0.934	8.6 (7)	7.4 (6)	0.772

^a^We performed 1:1 matching according to close gestational age (difference was ≤3 days) and birth weight (difference was ≤400 g)

### The effect of probiotics on the deterioration of NEC between the two groups

Before matching, we found that the rate of deterioration of NEC from stage I to II/III in the probiotic treatment group was similar to that in the group without probiotic treatment (23.1% [25/108] vs 26.0% [32/123], *P* = 0.614). After matching, the rate of deterioration of NEC between the group with probiotic treatment and the group without probiotic treatment still had no significant difference (21.0% [17/81] vs 27.2% [22/81], *P* = 0.358). For those infants who received probiotic treatment, no significant difference in the administration time of probiotics was found between the stage I group (*n* = 64) and the stage ≥II group (*n* = 17) (9[7–14.75] vs 12[6.5–23], *P* = 0.362).

### The risk factors related to deterioration of NEC

On the basis of the above findings, an interesting question was put forth regarding which factors may have contributed to the deterioration from stage I to II/III. We used the matched cases to design a case-control study to find those risk factors. Perinatal factors, complications and treatment therapy were compared between the stage I group (*n* = 123) and the stage ≥II group (*n* = 39), and none of the perinatal factors and baseline characteristics were significantly associated with NEC progression (Table 4 and Additional file 1: Table S2). However, sepsis (*P* = 0.021) after NEC was significantly related to the deterioration of NEC (Table 5). These statistically significant variables were tested again by logistic regression analysis, and sepsis (OR: 2.378, 95% CI: 1.005–5.628, *P* = 0.049) after NEC was identified as the independent risk factor for stage I NEC deteriorating to stage II/III NEC.

**Table 4:** The treatment protocols between stage I and ≥ II NEC infants after matching

Variables	Stage I (*n* = 123)	≥ Stage II (*n* = 39)	*P*
Gastrointestinal decompression, %(n)	57.7 (71)	79.5 (31)	0.014
Duration of gastrointestinal decompression, d	4 (2–6)	3 (2–6)	0.817
Blood exchange transfusion, %(n)	0.8 (1)	0 (0)	1.000
Red blood cell support, %(n)	23.6 (29)	20.5 (8)	0.691
Platelet support, %(n)	13.8 (17)	10.3 (4)	0.564
Plasma or cryoprecipitate support, %(n)	13.8 (17)	12.8 (5)	0.874
Intravenous immunoglobulin, %(n)	42.3 (52)	38.5 (15)	0.673
Albumin support, %(n)	24.4 (30)	23.1 (9)	0.867
Dopamine support, %(n)	7.3 (9)	10.3 (4)	0.802

We performed 1:1 matching according to close gestation age (difference was ≤3 days) and birth weight (difference was ≤400 g)

**Table 5:** Comparison of complications between infants with NEC stage I and those with ≥II after matching

Variables	Stage I (*n* = 123)	≥ Stage II (*n* = 39)	*P*
Neonatal respiratory syndrome, %(n)	8.1 (10)	2.6 (1)	0.402
Apnea, %(n)	4.1 (5)	7.7 (3)	0.626
Respiratory failure, %(n)	15.4 (19)	12.8 (5)	0.687
Pulmonary hemorrhage, %(n)	7.3 (9)	2.6 (1)	0.488
Sepsis, %(n)	30.9 (38)	51.3 (20)	0.021
Septic shock, %(n)	4.1 (5)	2.6 (1)	1.000
Bacterial meningitis, %(n)	5.7 (7)	5.1 (2)	1.000
Congenital heart disease, %(n)	46.3 (57)	48.7 (19)	0.796
Anemia, %(n)	69.9 (86)	64.1 (25)	0.496
Coagulation disorder, %(n)	59.3 (73)	56.4 (22)	0.745
Thrombocytopenia, %(n)	25.2 (31)	17.9 (7)	0.352
Cold injury syndrome, %(n)	3.3 (4)	5.1 (2)	0.957
Hypoproteinemia, %(n)	52.0 (64)	46.2 (18)	0.522

We performed 1:1 matching according to close gestational age (difference was ≤3 days) and birth weight (difference was ≤400 g)

## Discussion

There have been many reports that have evaluated probiotic administration for the prevention of NEC. Many previous reports of probiotics found that the use of probiotics was beneficial for the prevention of severe NEC. To our knowledge, data have rarely been specifically focused on whether probiotics prevent the deterioration of NEC from stage I to II/III in infants. In the present study, we found that probiotics could not prevent the deterioration of NEC from stage I to II/III in infants.

Breastfeeding has a protective effect against NEC [13]. Studies have found that human milk is not sterile, and up to 200 different bacterial species have been found in human milk [14]. One study involving the microbial detection of milk samples from healthy women collected at three different time points showed nine bacterial genera were present in all samples but in different concentrations among the subjects [14]. This finding suggests that milk microbes from each mother are optimized for the health of her own infant, and breast milk of healthy women is a source of commensal bacteria in the infant gut [15]. Therefore, the administration of large-scale industrially produced probiotics regardless of the NEC infants’ individual characteristics of intestinal microbes may not reduce the deterioration of NEC.

There are currently no unified standards for access schemes detailing routine use in the prevention of NEC. Probiotic type, dosage and administration timing may affect the influence of probiotics on the incidence of NEC, and controversial findings of the influence of probiotics on the incidence of NEC have been published [16–18]^.^ One guideline suggested that a daily dose of 3 × 10^9^ cfu/day may be appropriate for neonates of less than 32 weeks gestation [16]. Evidence indicates that to be functional, probiotics have to be viable and at sufficient dosage levels, typically 10^6^ to 10^7^ cfu/g of product, and many probiotic products have the same dose of bacteria [16]. It is not clear whether 10^6^ to 10^7^ (cfu)/g of product is effective in preventing the deterioration of NEC from stage I to II/III. Furthermore, given that the dose of 10^6^ cfu used in the present study was much lower than other studies (10^7^~10^10^ cfu) [17, 18], the conclusion should be treated with caution.

Early initiation of enteral feeding after NEC may have a beneficial effect on the recovery of the intestinal mucosa [19]. In contrast, late initiation of enteral feeding may lead to mucosal villous atrophy [19, 20]. There is a lack of consensus among surgeons and neonatologists regarding the optimal feeding strategy after an NEC diagnosis to prevent its recurrence. Most textbooks suggest bowel rest for 7 to 10 days, but there is a lack of clinical evidence to support this recommendation [21]. Initiating early enteral feeding within 5 days of NEC diagnosis (Bell stage II or above) is not associated with adverse outcomes, including NEC recurrence [22]. There was also no guideline regarding the initiation of feeding for infants with NEC Bell stage I.

There are some limitations in this study, including the errors and biases inherent to the nature of a retrospective study. In the present study, we performed 1:1 matching by using similar gestational age and birth weight, and it led to a loss of approximately 30% of sample capacity. Therefore, the overall sample capacity of the study was relatively small.

## Conclusions

In summary, we found that probiotics might not prevent the deterioration of NEC, but this conclusion should be treated with caution.

## Additional file

Additional file 1: **Table S1.** The complications of NEC infants treated (with/without) probiotics in this study. **Table S2.** Comparison of baseline information between infants with NEC stage I and those with ≥ II. (DOCX 20 kb)
