# Supplementary material for: Probiotics may not prevent the deterioration of necrotizing enterocolitis from stage I to II/III
Source: BMC Pediatr. 2019 Jun 8;19:185. doi: 10.1186/s12887-019-1524-5 (PMC6556034; doi:10.1186/s12887-019-1524-5)
Supplement: Supplementary file 1 — Table S1. The complications of NEC infants treated (with/without) probiotics in this study. Table S2. Comparison of baseline information between infants with NEC stage I and those with ≥ II. (DOCX 20 kb) [file 12887_2019_1524_MOESM1_ESM.docx]

| Table S1. The complications of NEC infants treated (with/without) probiotics in this study | | | | | | | |
| --- | --- | --- | --- | --- | --- | --- | --- |
| Variables | Total (n=231) | Before matching | | | After matching | | |
|  |  | With (n=108) | Without (n=123) | *P* | With (n=81) | Without (n=81) | *P* |
| Cold injury syndorme, %(n) | 3.9(9) | 4.6(5) | 3.3(4) | 0.842 | 3.7(3) | 3.7(3) | 1.000 |
| hemolytic disease, %(n) | 10.0(23) | 13.0(14) | 7.3(9) | 0.153 | 13.6(11) | 9.9(8) | 0.464 |
| Hepatic dysfunction, %(n) | 12.6(29) | 15.7(17) | 9.8(12) | 0.171 | 13.6(11) | 6.2(5) | 0.114 |
| Cholestasis, %(n) | 9.1(21) | 6.5(8) | 12.0(13) | 0.144 | 8.6(7) | 8.6(7) | 1.000 |
| Hypoproteinemia, %(n) | 51.9(120) | 54.6(59) | 49.6(61) | 0.445 | 55.6(45) | 45.7(37) | 0.209 |
| Hyperlactacidemia, %(n) | 13.9(32) | 14.8(16) | 13.0(16) | 0.692 | 14.8(12) | 16.0(13) | 0.828 |
| Hypoglycemia, %(n) | 17.3(40) | 21.3(23) | 13.8(17) | 0.134 | 19.8(16) | 12.3(10) | 0.199 |
| Hyperglycemia, %(n) | 3.0(7) | 2.8(3) | 3.3(4) | 1.000 | 3.7(3) | 3.7(3) | 1.000 |
| Hypokalemia, %(n) | 32.5(95) | 37.0(40) | 28.5(35) | 0.165 | 38.3(31) | 28.4(23) | 0.182 |
| Hyperkalemia, %(n) | 19.0(44) | 18.5(20) | 19.5(24) | 0.848 | 19.8(16) | 16.0(13) | 0.539 |
| Hypochloremia, %(n) | 13.0(30) | 14.8(16) | 11.4(14) | 0.439 | 14.8(12) | 13.6(11) | 0.822 |
| Hyponatremia, %(n) | 15.6(36) | 13.0(14) | 17.9(22) | 0.303 | 14.8(12) | 21.0(17) | 0.305 |
| Hypomagnesemia, %(n) | 9.5(22) | 9.3(10) | 9.8(12) | 0.898 | 9.9(8) | 8.6(7) | 0.786 |

| Table S2. Comparison of baseline information between infants with NEC stage I and those with ≥ II | | | | | | |
| --- | --- | --- | --- | --- | --- | --- |
| Variables | Before matching | | | After matching | | |
|  | Stage Ⅰ (n=174) | ≥ Stage Ⅱ (n=57) | *P* | Stage Ⅰ(n=123) | ≥ Stage Ⅱ (n=39) | *P* |
| Gestational age, IQR, wks | 38 (35.11-39.71) | 37 (33.5-39.71) | 0.178 | 37.86 (35.14-39) | 37.86 (34.43-39.71) | 0.995 |
| Birth weight, (M±SD),g | 2900 (2100-3342.5) | 2700 (1920-3250) | 0.217 | 2641.89±715.89 | 2669.36±725.76 | 0.835 |
| Premature, %(n) | 36.2(63) | 49.1(28) | 0.083 | 37.4(46) | 43.6(17) | 0.490 |
| The age of onset, IQR, d | 3.67 (1.09-10.03) | 4.25 (1.13-19.59) | 0.166 | 3.44 (1.08-9.83) | 6 (1.33-20) | 0.093 |
| Natural delivery, %(n) | 44.3(77) | 35.1(20) | 0.224 | 45.5(56) | 35.9(14) | 0.290 |
| PROM>18 h, %(n) | 4.6(7) | 7.0(4) | 0.711 | 4.1(5) | 5.1(2) | 1.000 |
| Amniotic fluid contamination, %(n) | 13.2(23) | 14.0(8) | 0.875 | 13.0(16) | 17.9(7) | 0.441 |
| Asphyxia, %(n) | 11.5(20) | 8.8(5) | 0.566 | 13.8(17) | 7.7(3) | 0.463 |
| Infants of diabetic mother, %(n) | 2.9(5) | 1.8(1) | 1.000 | 3.3(4) | 2.6(1) | 1.000 |
| Pregnancy-included hypertension, %(n) | 6.3(11) | 3.5(2) | 0.639 | 8.1(10) | 2.6(1) | 0.402 |
| ICP, %(n) | 1.7(3) | 3.5(2) | 0.780 | 0.8(1) | 5.1(2) | 0.144 |
| Antenatal corticosteroids, %(n) | 2.9(5) | 5.3(3) | 0.661 | 3.3(4) | 5.1(2) | 0.957 |
| PROM= prolonged rupture of membranes, ICP =intrahepatic cholestasis of pregnancy | | | | | | |
